# How useful is the current species recognition concept for the determination of true morels? Insights from the Czech Republic

**DOI:** 10.3897/mycokeys.52.32335

**Published:** 2019-05-09

**Authors:** Irena Petrželová, Michal Sochor

**Affiliations:** 1 Centre of the Region Haná for Biotechnological & Agricultural Research, Crop Research Institute, Dept. of Genetic Resources for Vegetables, Medicinal & Special Plants, Šlechtitelů 29, Olomouc-Holice, CZ-78371, Czech Republic Centre of the Region Haná for Biotechnological & Agricultural Research Olomouc Czech Republic

**Keywords:** GCPSR, Mel-39, *
Morchella
*, multigene analysis, phylospecies, species concept

## Abstract

The phylogentic diversity of the genus *Morchella* has only been sporadically studied in Central Europe. In this study, a molecular taxonomic revision of the *Morchella* species of the Czech Republic was performed using available fungarium specimens, fresh collections, and axenic cultures. Molecular phylogenetic analyses based on either ITS or five-locus (ITS, LSU, *RPB1*, *RPB2*, and *EF-1α*) sequencing and the application of principles of the genealogical concordance phylogenetic species recognition (GCPSR) have revealed the occurrence of 11 phylogenetic species in the region, but only six of them could be assigned unequivocally to the previously published phylospecies: Mel-3 (*M.semilibera*), Mel-10 (*M.importuna*), Mel-19 (*M.eohespera*), Mes-4 (*M.americana*), Mes-5 and Mes-8 (*M.esculenta*). One lineage was identified as a new phylospecies and is designated as Mel-39. Four lineages grouped together with two or more previously published phylospecies: Mel-13/26 (*M.deliciosa*), Mel-15/16 (*M.angusticeps* / *M.eximioides*), Mel-20/34 (*M.purpurascens*), and Mel-23/24/31/32 (*M.pulchella*). Our phylogenetic analyses and literature review shed light on the pitfalls of current molecular taxonomy of morels and highlight the ambiguities of present species recognition concepts. The main source of the problems seems to be rooted in the application of different methods (multigene vs single-gene sequencing, phenotypic determination) and approaches (monophyly vs paraphyly, the application or not of GCPSR, degree of differentiation between accepted species, etc.) by various authors for the delimitation of new phylospecies. Therefore, we propose five criteria for distinguishing new phylospecies in the genus *Morchella* based on molecular data, and recommend a more conservative approach in species delimitation.

## Introduction

True morels (genus *Morchella* Dill. ex Pers.: Fr.) are edible ascomycete fungi characterized by a honeycomb appearance and a spring fruiting (at least in the temperate zone), with the exception of a couple of autumnally occurring species (e.g. Masaphy et al. 2009; Matočec et al. 2014; Taşkın et al. 2015). Morels are amongst the most highly prized fungi worldwide, not only for their taste, but also for their nutritional value and medicinal properties (Tietel and Masaphy 2018). The genus is distributed worldwide. However, recent molecular phylogenetic studies suggest that the individual species exhibit high continental endemism and provincialism in the Northern Hemisphere (O’Donnell et al. 2011), and approximately 20 species have been recorded on more than one continent (Taşkın et al. 2010, 2012, 2015; O’Donnell et al. 2011; Du et al. 2012a; Pildain et al. 2014; Richard et al. 2015; Loizides et al. 2016, 2017; Yatsiuk et al. 2016; Loizides 2017). The highest species diversity of true morels is concentrated in Europe and West Asia, East Asia (mainly China), and North America (Du et al. 2015; Richard et al. 2015). One of the worldwide diversity hotspots is the Mediterranean and adjacent regions, particularly Turkey (with more than 20 species; Taşkın et al. 2010, 2012) and Cyprus (11 species; Loizides et al. 2016).

For taxonomists and field mycologists, true morels are known as a very intricate genus. Three easily distinguishable evolutionary lineages (clades) and three corresponding sections are currently recognized: (i) the basal Rufobrunnea Clade (sect. Rufobrunnea, or “white morels”), (ii) the Elata Clade (sect. Distantes, or “black morels”) and (iii) the Esculenta Clade (sect. Morchella, or “yellow morels”). Nevertheless, the lack of discriminatory micromorphological characters and in some cases extreme macromorphological variability and/or plasticity have complicated the delimitation and characterization of species. Therefore, phenotypic characters have often been complemented with the geographic occurrence and/or ecology in recent studies, especially putative associations with particular trees or shrubs, which can sometimes be taxonomically informative (Clowez 2012; Kuo et al. 2012; Clowez et al. 2014; Loizides et al. 2015; Loizides 2017; Baroni et al. 2018). It is supposed that black morels may be either mycorrhizal or saprotrophic, some of them being obligate or facultative pyrophiles (Loizides 2017). Yellow morels are considered to be exclusively mycorrhizal (Li et al. 2013) and, thus, are probably more tightly associated with their autotrophic partners.

Current taxonomic and systematic studies on morels are mostly based on multilocus DNA sequencing (Taşkın et al. 2010, 2012; O’Donnell et al. 2011; Du et al. 2012a; Richard et al. 2015), which allows for species delimitation and phylogeny inference. By employing sequence data from four to five nuclear genomic loci (nuc 28S rDNA [LSU], RNA polymerase largest [*RPB1*] and second largest subunit [*RPB2*], translation elongation factor 1-alpha [*EF-1α*], and for particular groups also nuc rDNA ITS1-5.8S-ITS2 [ITS]) and principles of genealogical concordance phylogenetic species recognition (GCPSR; Taylor et al. 2000), O’Donnell et al. (2011) distinguished 41 phylogenetic species (phylospecies) in three major clades across the globe: 24 in the Elata Clade, 16 in the Esculenta Clade and one species in the Rufobrunnea Clade. In parallel or later, many new phylospecies were distinguished by several authors, who did not always utilize the multigene approach and/or basic phylogenetic principles (such as monophyly), not to speak of GCPSR (Taşkın et al. 2010, 2012; Du et al. 2012a; Elliott et al. 2014; Pildain et al. 2014; Loizides et al. 2016; Voitk et al. 2016a). Because binominal names can be unambiguously assigned to only a part of the phylospecies, they are usually (but not by all authors) denoted by a clade abbreviation followed by an Arabic number (Mel-1 to Mel-38 for the Elata Clade and Mes-1 to Mes-28 for the Esculenta Clade; Taşkın et al. 2010; O’Donnell et al. 2011). In total, 76 distinct (phylo)species have so far been recognized within the genus *Morchella* worldwide, including 25 species recorded in continental Europe (O’Donnell et al. 2011; Du et al. 2012b; Taskin et al. 2012; Clowez et al. 2014, 2015; Richard et al. 2015; Yatsiuk et al. 2016; Baroni et al. 2018). However, data on the *Morchella* species diversity from Central Europe are lacking.

In the Czech Republic and former Czechoslovakia, studies on Ascomycota have a long tradition and popularity, and several *Morchella* species were even described from the Czech territory (Krombholz 1831–1834; Velenovský 1934; Smotlacha 1947, 1952; Šebek 1973). However, the available literature on morels is rather confusing and far from being clear. With the exception of a few Czech specimens included in the worldwide molecular studies (O’Donnell et al. 2011) and a single study by Ondřej et al. (2011), who employed the sequencing of the 5.8S-ITS2 region and AFLP markers to characterize the diversity of bark mulch morels, all previous studies were limited to phenotypic and ecological species identification. The following species are usually reported as occurring in the Czech Republic: *M.angusticeps* Peck, *M.conica* Pers., *M.crassipes* (Vent.) Pers., *M.elata* Fr., *M.esculenta* (L.) Pers., *M.pragensis* Smotl., *M.semilibera* DC., and *M.vulgaris* (Pers.) Gray (Holec et al. 2012; Mikšík 2015). However, *M.angusticeps* is currently used only for the probably endemic American species, *M.conica* is considered illegitimate (Richard et al. 2015), collections formerly treated as *M.crassipes* were recently determined to be several Esculenta Clade species on the basis of sequencing data (Du et al. 2012b; Richard et al. 2015), the taxonomical status of *M.elata* is still unresolved (Richard et al. 2015), and *M.pragensis* is a rather mysterious species that also remains phylogenetically and taxonomically unresolved (see below).

We therefore performed a detailed molecular taxonomic revision of true morels in the Czech Republic on the basis of recent collections and available fungarium specimens within the framework of phylogenetic species recognition as initiated by O’Donnell et al. (2011) and followed by a number of other authors. However, our analysis has failed to discriminate between several published phylospecies, questioning the accuracy and consistency of currently applied species recognition methods. As a result, a revised phylogenetic species concept is proposed and suggestions regarding the criteria for the recognition of morel species are presented.

## Materials and methods

### Sampling and culturing

Our sampling aimed at covering the territory of the Czech Republic, and to a lesser extent adjacent parts of Slovakia, using two sources of material. First, for cultivation of axenic cultures and subsequent molecular analysis, 66 fresh specimens of *Morchella* that originated from our own recent collections or were provided by collaborating mycologists in 2008–2018, were used. Fruiting bodies from each micro-locality (unless significantly different in appearance) were considered as a single specimen and a single fruiting body was usually used for cultivation and/or analysis. However, at nine localities two to twelve mulch morels ascomata were analyzed to assess species diversity within the mulch beds (Suppl. material 1, Table S1). Cultures were derived either from the spore prints or from the inner tissues of ascomata transferred into Petri dishes with a malt extract glucose agar medium (MEGA; 10 g/L malt extract, 5 g/L glucose, 15 g/L agar) supplemented with chloramphenicol (100 mg/L). Cultivation was carried out in the dark at 18–20 °C. Axenic cultures of the obtained strains maintained on a rye grain substrate are available as a part of the Collection of Edible & Medicinal Macromycetes (CEMM) maintained within the framework of The Czech National Programme on Conservation & Utilization of Microbial Genetic Resources Important for Agriculture (http://www.vurv.cz/cspp/mikroorganismy/Edible and Medicinal macromycetes.html) at the Crop Research Institute (https://www.vurv.cz). For DNA extraction, the mycelium or sclerotia of individual strains were sampled from jars with the rye grain spawn prepared by the inoculation of pre-soaked and sterilized rye grains with pieces of agar covered with morel mycelium. From 16 specimens (including five samples from Slovakia) in which the derivation of axenic cultures was unsuccessful (marked as n.m.d. in Suppl. material 2, Table S2) DNA was extracted directly from fresh-frozen or dried pieces of ascomata.

Secondly, for the DNA analysis only, 377 morel specimens in total were obtained from the selected Czech public herbaria and one private fungarium, of which 203 were successfully analyzed (abbreviations according to Thiers 2018): BRNM: 73 specimens, CB: 50 specimens, CHOM: 23 specimens, HR: 24 specimens, LIT: 12 specimens, PL: six specimens, PRC: three specimens, and Vavřinec Klener’s private fungarium: 12 specimens). The specimens were collected between the years 1950 and 2018. For details see Suppl. material 2, Table S2.

### Molecular analysis

Total genomic DNA was extracted from ca < 10 mg of dry fruiting body or an equivalent amount of the fresh mycelium culture or sclerotia by the CTAB method (Doyle and Doyle 1987). The ITS locus was amplified and sequenced in all the studied accessions using the ITS1F (Gardes and Bruns 1993) and ITS4 primers (White et al. 1990) or, in the case of old specimens with fragmented DNA, either with ITS1F and ITS2, or ITS3 and ITS4 (White et al. 1990). Subsequently, at least two representative accessions per detected phylospecies (with respect to the detected variation in ITS) and approximately six accessions within species-rich complexes were selected for further sequencing. *RPB1* was amplified and sequenced with the gRPB1-A and fRPB1-C primers (Matheny et al. 2002), *RPB2* with the fRPB2-7cF (Liu et al. 1999) and RPB2-3053r primers (Reeb et al. 2004), *EF-1α* with the EF-526F and EF1567R primers (Rehner and Buckley 2005), and domains D1 and D2 of 28S rDNA (LSU) with the NL1 and NL4 primers (O’Donnell 1993). All the PCRs were performed in 20-μL reaction mixtures with Kapa polymerase (Kapa Biosystems, Massachusetts, USA) and a touchdown protocol with an annealing temperature of 61–56 °C in the first six cycles and 56 °C in the following 37 cycles. The PCR products were purified by precipitation with polyethylene glycol (10% PEG 6000 and 1.25 M NaCl in the precipitation mixture) and sequenced by the Sanger method at Macrogen Europe (The Netherlands).

### Data analysis

Sequences were edited and aligned in Geneious 7.1.7. (Biomatters, New Zealand) using the MAFFT plugin and deposited in NCBI GenBank under the accession numbers MH982584–MH983000. Alleles of the ITS locus were distinguished on the basis of single nucleotide polymorphisms and compared to the publicly available sequences. Bayesian phylogeny inference in MrBayes 3.2.4 (Ronquist et al. 2012) with 10^7^ generations, sampling every 1000^th^ tree, in two independent runs, each with six chains, 50% burn-in and a temp parameter of 0.01 was used for the preliminary assignment to the published phylogenetic species. As a reference database, previously published sequence data from one to two accessions per phylospecies were selected in order to cover the total species richness and the widest possible intraspecific variation. Multilocus sequences from the selected accessions were concatenated and ambiguously aligned parts (ITS1 in the Esculenta Clade dataset) and ends of the sequences with many missing data were discarded. Bayesian phylogeny inference for concatenated data was computed in MrBayes with 20 million generations, sampling every 1000^th^ tree, in two independent runs, each with four chains, and the first 10 million generations (50%) were excluded as burn-in. A substitution model for each locus was determined in Partitionfinder 2.1.1 (Lanfear et al. 2017) using the corrected AIC (AICc) and a greedy search, and partitions were subsequently set in MrBayes according to the loci.

## Results

Out of a total of 377 fungarium specimens of different ages (mostly < 50 years), we were able to obtain at least a partial informative ITS sequence for 211 specimens, of which eight specimens from Mel-19 (*M.eohespera* Beug, Voitk & O‘Donnell), Mel-20/34 (*M.purpurascens* (Boud.) Jacquet), or Mel-23/24/31/32 (*M.pulchella* Clowez & F. Petit) could not be determined because of the insufficient sequence length and therefore they were excluded from the analyses. The success rate only partly corresponded to the age, as even exsiccata that were several decades old contained relatively well-preserved DNA and many specimens that were one or a few years old had very degraded DNA, particularly if the fruiting body had been attacked by larvae or dried slowly (data not shown). ITS seems to be insufficient for distinguishing between Mel-19 and Mel-20/34, which differ in a single SNP in ITS2 closely adjacent to 5.8S rDNA. This SNP may be uninformative on a wider geographic scale, as the Mel-19 (*M.eohespera*) variant was observed in some published sequences of Mel-20 (*M.purpurascens*). However, because this SNP was stable in our data set, we used it for the determination of specimens analyzed solely by ITS. Though, our method of preliminary identification based on ITS proved to be successful and robust for most fresh and fungarium specimens (Suppl. material 3, Fig. S1).

3766 bp and 3464 bp alignments were constructed from 102 and 39 specimens for the Elata Clade and the Esculenta Clade, respectively, including a representative set of the published sequences (Suppl. material 4, supplementary data). According to our expectations, ITS revealed the highest variability (ca 65% and 67% of variable sites in the Elata and the Esculenta Clade, respectively; see also Suppl. material 5, Table S3) and therefore proved to be the most suitable for screening of the phylogenetic diversity of the whole sample set. The least polymorphic locus was LSU (11% and 7%), *EF-1α* exhibited 27% and 18%, *RPB1* 25% and 12%, and *RPB2* 21% and 12% of polymorphic sites in the Elata and the Esculenta Clade, respectively.

**Figure 1.**
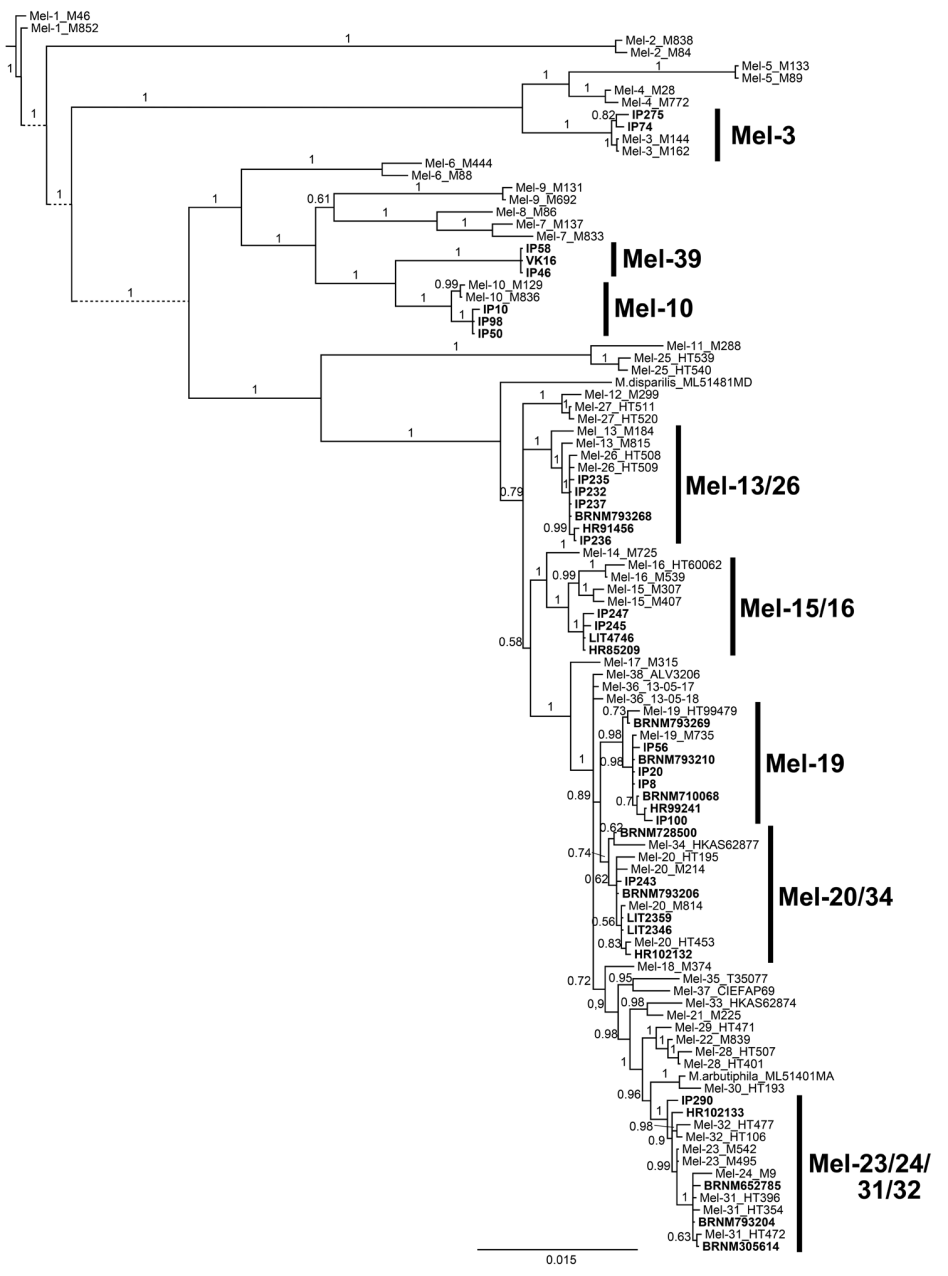
Bayesian phylogeny inference tree based on five-gene concatenated alignment from selected accessions of the Elata Clade. Posterior probabilities (PP) are shown above branches, splits with PP < 50% were collapsed.

**Figure 2.**
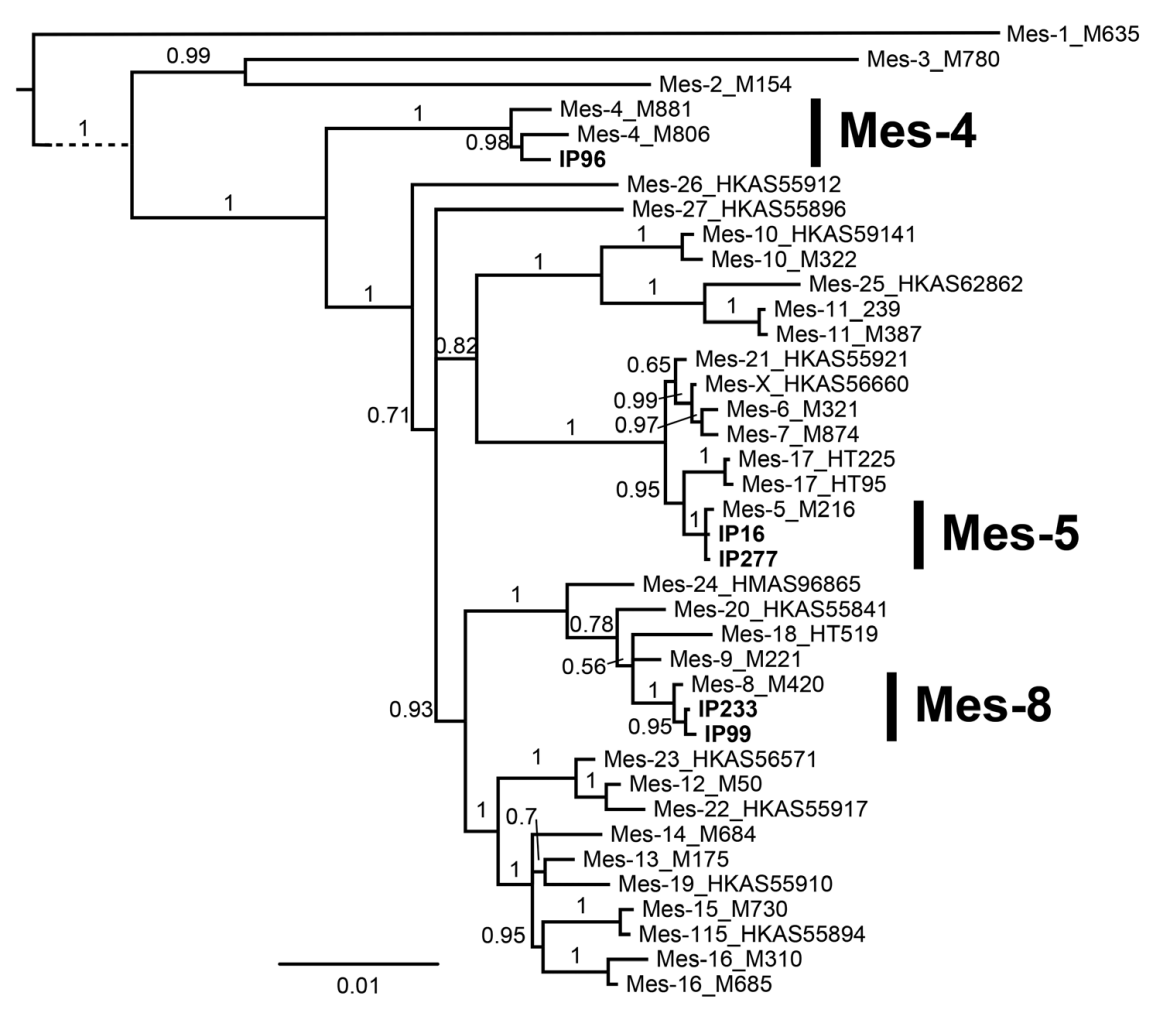
Bayesian phylogeny inference tree based on five-gene concatenated alignment from selected accessions of the Esculenta Clade. Posterior probabilities (PP) are shown above branches, splits with PP < 50% were collapsed.

Bayesian analysis of the multilocus data placed all of the Czech specimens into a highly supported branch together with other specimens that were analyzed, but only six of the lineages contained a single published species and could be determined unambiguously: Mel-3 (*M.semilibera*), Mel-10 (*M.importuna* M. Kuo, O‘Donnell & T.J. Volk), Mel-19 (*M.eohespera*), Mes-4 (*M.americana* Clowez & Matherly), Mes-5 and Mes-8 (*M.esculenta*) (Figs 1, 2). Mel-19 was separated from Mel-20/34 (*M.purpurascens*) in *EF-1α* and multilocus analysis, yet rather weakly diverged (Suppl. material 5: Table S3), and appeared polyphyletic at *RPB2* (Suppl. material 3, Fig. S1). Four specimens that were used for multigene analysis and six specimens analyzed for ITS only formed a basal lineage to Mel-15 (*M.angusticeps*) and Mel-16 (*M.eximioides* Jacquet.). Although this lineage is highly supported in all analyses (Fig. 1, Suppl. material 3, Fig. S1), its genetic distance from Mel-15 and Mel-16 is only 0–9 SNP’s at every locus (Suppl. material 5, Table S3). Six specimens (of which three were included in the multigene analysis) formed a well-separated and highly supported clade sister to Mel-10 (*M.importuna*) in the multigene analysis and all the single-gene analyses (posterior probability 1.0, or 0.98 for ITS; Fig. 1, Suppl. material 3, Fig. S1). The genetic distance of this clade from Mel-10 was 12–21 SNP’s at most loci and no variation was detected within the clade (Suppl. material 5, Table S3). These six specimens were designated as a new phylospecies, annotated “Mel-39”. Other specimens that were studied were intermixed within the clusters of two or four previously recognized phylospecies and could not be assigned unambiguously to a single one of them because of the high intra-specific variation (autapomorphies) and the lack of shared polymorphism (synapomorphies).

**Figure 3.**
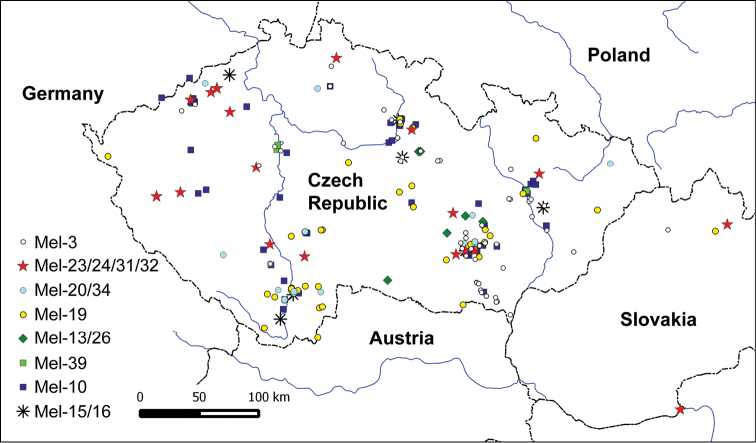
Distribution of the Elata Clade phylospecies in the Czech Republic (and Slovakia) based on identification by ITS or multi-gene sequencing, or phenotypic identification (in the case of Mel-3). For details see Supplementary Table 2.

**Figure 4.**
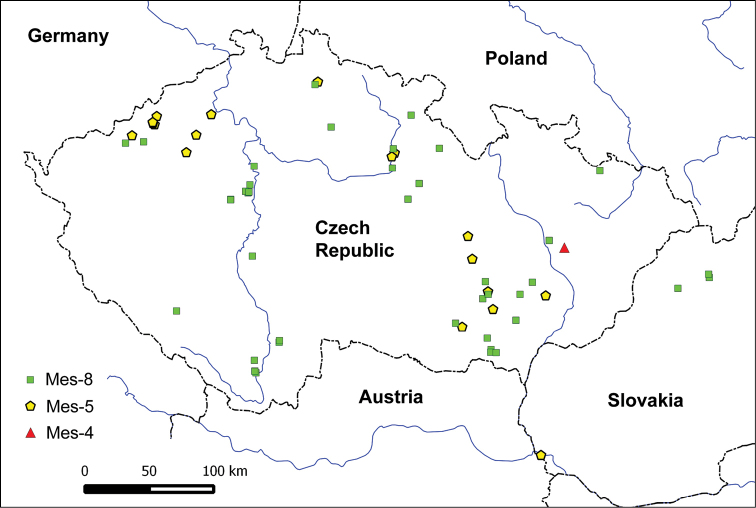
Distribution of the Esculenta Clade phylospecies in the Czech Republic (and Slovakia) based on identification by ITS or multi-gene sequencing. For details see Supplementary Table 2.

Geographic mapping of the analyzed accessions did not reveal any clear patterns in the phylospecies distribution (Figs 3, 4). Every species was distributed in all the lowland to lower montane areas of the Czech Republic; only Mes-5 was not detected in the southern half of Bohemia and northern parts of Moravia (Fig. 4), and Mel-19 (*M.eohespera*) is underrepresented in the north-western half of Bohemia (Fig. 3), which may, however, only reflect the density of sampling in these regions.

## Discussion

On the basis of the five-gene sequencing of 41 collections and ITS sequencing of a further 228 collections, we distinguished 11 phylogenetic lineages occurring in the Czech Republic. Only six lineages clustered tightly to a single one of the published phylospecies, whereas four lineages grouped together with two or more previously published species. One lineage was unique, without close affinity to any known phylospecies.

The concept of the phylogenetic species recognition in the genus *Morchella* was developed by O’Donnell et al. (2011) on the basis of multi-gene sequencing of global set of *Morchella* specimens using the principles of genealogical concordance phylogenetic species recognition (GCPSR; Taylor et al. 2000). *Morchellarufobrunnea* Guzmán & F. Tapia, Mel-1 to Mel-24 and Mes-1 to Mes-16 were distinguished first. In parallel, *M.anatolica* Işıloğlu, Spooner, Allı & Solak (the Rufobrunnea Clade), Mes-17, Mes-18 and Mel-25 to Mel-32 were distinguished from Turkey (Işiloğlu et al. 2010; Taşkın et al. 2010, 2012). In China, Du et al. (2012a) recognized eleven new phylospecies (Mel-33 and Mel-34 and Mes-19 to Mes-27). Mel-35 was designated to the Australian species *M.australiana* T.F. Elliott, Bougher, O‘Donnell & Trappe (Elliott et al. 2014), Mel-36 (named as *M.laurentiniana* Voitk, Burzynski, O’Donnell) was described from Canada (Voitk et al. 2016a), Mel-37 from Argentina (Pildain et al. 2014), and four new species (Mes-28, Mel-38, *M.disparilis* Loizides & P.-A. Moreau and *M.arbutiphila* Loizides, Bellanger & P.-A. Moreau, both without phylospecies designation) were described from Cyprus (Loizides et al. 2016). From Spain, *M.castaneae* L. Romero & Clowez and *M.palazonii* Clowez & L. Romero (both without a phylospecies designation) were described by Clowez (2012) and Clowez et al. (2015) based on morphology and ITS sequencing. Most recently, *M.kaibabensis* Beug, T.A. Clem. & T.J. Baroni and *M.peruviana* S.A. Cantrell, Lodge, T.J. Baroni & O’Donnell were described from Arizona and Peru (Baroni et al. 2018).

Two independent studies with descriptions of several new species were published in 2012 (Clowez 2012; Kuo et al. 2012; the former not reflecting previous molecular analyses, the latter with the aim of assigning Latin binomials to the unnamed phylospecies). However, the names proposed by Clowez (2012) have priority over those published for the same taxa by Kuo et al. (2012). A unified taxonomy for the known European and North American species was therefore later proposed by Richard et al. (2015), who performed a nomenclatorial revision, typification of some ambiguous names and synonymization of the names published by Clowez (2012) and Kuo et al. (2012). Of the phylospecies originally identified by O’Donnell et al. (2011), binominal names have so far been assigned to 18 Mel- species and nine Mes- species (Richard et al. 2015; Voitk et al. 2016a; Baroni et al 2018). To the phylospecies that were distinguished from Turkey by Taşkın et al. (2010, 2012), scientific names were assigned by Clowez et al. (2014), Richard et al. (2015), and Taşkın et al. (2016). Additionally, Richard et al. (2015) synonymized several different taxa described by Clowez (2012) under the priority name *M.vulgaris*, which corresponds to Mes-17. However, on the basis of seven nucleotide changes in ITS (mainly ITS1) and morphological and ecological observations, Loizides et al. (2016) separated Mes-17 corresponding to *M.dunensis* (Castañera, J.L. Alonso & G. Moreno) Clowez (incl. *M.andalusiae* Clowez & L. Romero) as a separate sister species to *M.vulgaris*, which was left without a phylospecies designation.

To summarize, 76 (phylo)species have so far been recognized in the genus worldwide. However, taxonomic concepts differ greatly in terms of both methods (multigene sequencing, single-gene sequencing or phenotypic observation) and approaches (monophyly vs paraphyly, GCPSR or not, degree of genetic/phenotypic differentiation between accepted species, minimal number of collections, application of binominals or phylospecies designations, etc.). Together with the relatively high number of recent publications on morels, this conceptual diversity has led to much confusion and many contradictions. Therefore, on the basis of our data and a literature review, we discuss here some of the conceptual problems with *Morchella* species recognition and suggest basic rules that may prevent the introduction of unnecessary new taxa in the future.

### Suggestions for a sustainable morel taxonomy

The phylospecies concept of O’Donnell et al. (2011) was based explicitly on two main criteria. First, species were recognized if they were resolved as reciprocally monophyletic in at least one of the individual (i.e., single-locus) phylogenies and in the combined dataset (let us call this the “criterion of monophyly”), and second, if their genealogical exclusivity was not contradicted by analyses of any individual data partition (“genealogical criterion”). Understandably, none of these criteria can be fulfilled in species with a single collection. Therefore, the recognition of such species was based on a third criterion, i.e., genetic divergence from their sisters (“criterion of distinctness”). The three criteria are fully legitimate and intuitive. Nevertheless, whether they are met or not is always dependent on a particular dataset. After the addition of more collections (e.g., from different geographic regions), the criteria may cease to be fulfilled and the species thus become “illegitimate”. This seems to be the case of Mel-23 and Mel-24, which appeared to be reciprocally monophyletic and well differentiated originally, but several Czech specimens, as well as specimens previously determined as Mel-31 and Mel-32, share apomorphies with both of the lineages at each of the analyzed loci and, furthermore, exhibit several unique mutations. Their phylogenetic position therefore disrupts the clear distinctness of Mel-23 and Mel-24. At the same time, the Czech specimens do not form a separate lineage(s) and do not fulfill any of the three criteria (Fig. 1, Suppl. material 3, Fig. S1, Suppl. material 5, Table S3) and cannot be distinguished as separate phylospecies.

The criterion of monophyly and the genealogical criterion were not employed in many of the recent studies and were violated either consciously or because only one specimen was available. This is the case of, e.g., Mel-26 (*M.deliciosa* Fr.), Mel-31 (*M.pulchella*; see below), or Mel-33. Similarly, the criterion of distinctness seems to be considered only rarely and little attention seems to be paid to the genetic differentiation among newly distinguished phylospecies and the related ones in many studies. For instance, Mel-34 was distinguished as a separate lineage sister to Mel-20 (*M.purpurascens*) on the basis of a single specimen. Its genotype was similar or identical to Mel-20 at all the loci except *RPB1*, which provided an almost identical sequence to Mel-23/24/31/32 (*M.pulchella*; Suppl. material 3, Fig. S1, Suppl. material 5, Table S3, see also below). This discrepancy may represent a true biological signal, e.g. incomplete lineage sorting (Leliaert et al. 2014), but divergence between Mel-20 and Mel-23/24/31/32 is substantial (Fig. 1) and such an explanation is therefore questionable. This case illustrates how important it is to justify the distinction of new phylospecies by (i) the number of genetic differences at each locus and (ii) the inclusion of more than one sample in the analyses, which enables the effective exposure of base miscalling, erroneous alignment, PCR mutations, contaminations and other technical and processing errors that pose the high risk of introducing artifacts as new species (Thines et al. 2018). Beside the above-mentioned criteria, we therefore suggest that every newly distinguished phylospecies should be based on several (optimally three or more) different specimens (“criterion of minimal sampling”), and that it should differ from closely related species at most of the highly variable loci (in the case of morels, i.e., *EF-1α*, ITS, *RPB1*, *RPB2*) by at least one, but preferably more SNP’s that would be shared by all the individuals that are studied (“criterion of polygenic differentiation”). Although the latter criterion may be rather pragmatic and not fully reflect theoretical evolutionary processes at different loci, our analysis of average genetic differentiation shows that the closely related phylospecies differ at every locus by > 2 (but usually > 10) SNP’s (Suppl. matirial 5, Table S3). The criterion is therefore supported by empirical evidence and could also be useful for the potential recognition of new species in the future.

It is important to note that the proposed criteria should not be viewed as definite and insurmountable limits for taxonomy, but rather as a recommendation for cautiousness in introduction of new (phylo)species. Incomplete lineage sorting, hybridization, evolutionary stasis, and other factors may affect phylogenetic signal at each locus, particularly in recently diverged lineages (Mailund et al. 2014). Such closely related lineages may have already achieved reproductive isolation and segregated in distinct ecological or biogeographical compartments, they may even have acquired some diagnostic morphological traits, but DNA phylogenies may fail to assign them to (reciprocally) monophyletic clades (reviewed by Leliaert et al. 2014). Final taxonomic decision may therefore be influenced by stronger lines of evidence than (weak) patterns in DNA sequence variation. Nevertheless, as stressed by Carstens et al. (2013: 4369), taxonomic inferences should be conservative, “for in most contexts it is better to fail to delimit species than it is to falsely delimit entities that do not represent actual evolutionary lineages”.

### *Morchella* diversity in the Czech Republic with notes on taxonomy, nomenclature and ecology

**Figure 5.**
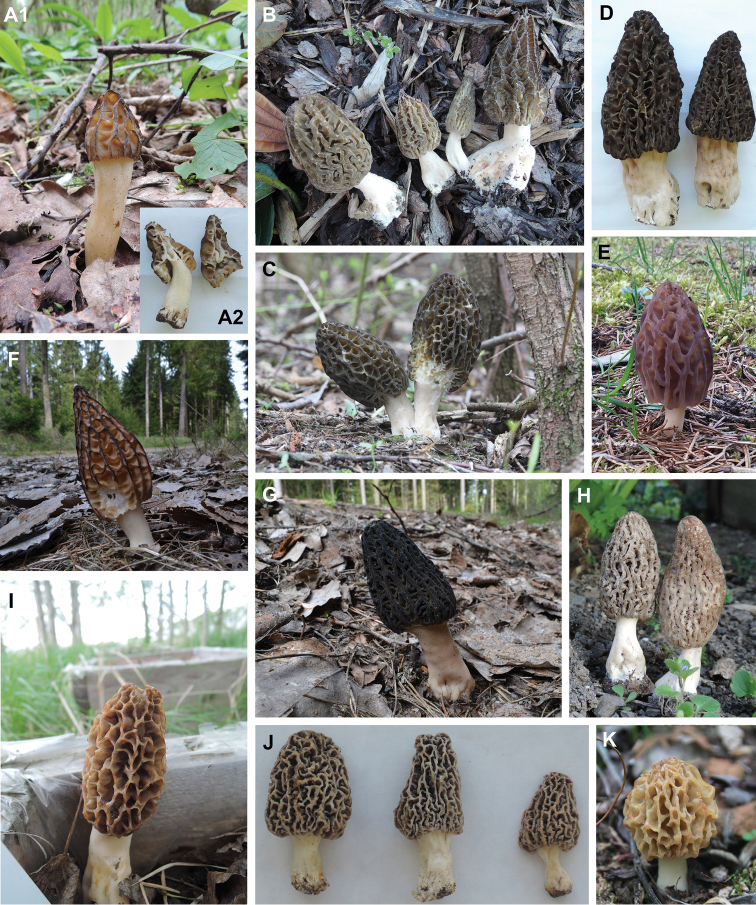
Examples of the fruiting bodies of *Morchella* phylospecies in the Czech Republic. **A1–2** Mel-3 (*M.semilibera*; A1. accession number VK13, A2. IP229) **B** Mel-10 (*M.importuna*; IP26) **C** Mel-13/26 (HR86151) **D** Mel-15/16 (IP245 and IP247) **E** Mel-19 (*M.eohespera*; HR99241) **F** Mel-20/34 (HR102132) **G** Mel-23/24/31/32 (HR102133) **H** Mel-39 (VK17) **I** Mes-4 (*M.americana*; IP297) **J** Mes-5 (IP350, IP351) **K** Mes-8 (*M.esculenta*; IP341) Photographers: Vavřinec Klener (**A1, H, K**); Irena Petrželová (**A2, B, D, I, J**); Jan Kramoliš (**F, G**); Dušan Bureš (**C, E**).

According to our analyses, the phylogenetic lineages of morels occurring in the Czech Republic are as follows (arranged by the phylospecies designations):

***Mel-3* (*M.semilibera* DC.; Fig. 5A).** Seventeen *Morchella* cultures or exsiccated specimens (one originating from Slovakia) were proven to be Mel-3, corresponding to *Morchellasemilibera* (syn. *Mitrophorasemilibera* (DC.) Lév., *Morchellagigas* (Batsch) Pers. or *M.hybrida* Pers.), which is in accordance with the previous phenotypic determination of the specimens. Ascomata were collected from mid-April to mid-May. As morphological features seemed to be highly reliable for the delimitation of this species (there is only the possibility of confusion with *Verpabohemica* (Krombh.) J. Schröt.), fungarium specimens were mostly not used for DNA analyses. However, collection data for 50 *M.semilibera* fungarium specimens were included on a map (Fig. 3) to demonstrate the species distribution in the Czech Republic. *Morchellasemilibera* is a widely distributed Eurasian species that had previously been recorded not only from the Czech Republic, but its occurrence was also confirmed molecularly from France, Germany, Italy, the Netherlands, Spain, Sweden, Turkey, and India (Taşkın et al. 2010, 2012; Kanwal et al. 2011; O’Donnell et al. 2011; Clowez 2012; Du et al. 2012b; Richard et al. 2015).

The Czech collections originated mostly from (semi-)natural habitats such as deciduous or, less frequently, mixed forests and floodplain forests (note that most of what are termed forests in Central Europe are semi-natural or completely artificial), groves, old fruit orchards, shrubs, or rocks. Only rarely was *M.semilibera* found in urban areas, e.g., in gardens, town parks, or also in ornamental beds, but we have no information as to whether there was bark mulch or not. The species appeared most frequently in association with *Fraxinus* spp., *Carpinusbetulus*, *Quercus* sp., *Acer* spp., *Prunus* spp. (especially *P.spinosa*), and cherry trees. According to the literature, *M.semilibera* often grows under *Fraxinusexcelsior* (Clowez 2012; Richard et al. 2015), and it was also found under *Malussylvestris*, *Castanea* sp., and *Populus* sp. (Taşkın et al. 2010, 2012; Richard et al. 2015). Judging by our recent collections and the representation of the species in herbaria, *M.semilibera* seems to be one of the most common *Morchella* species and is widespread in lowland areas of the Czech Republic (Fig. 3). However, in the national red list of macromycetes it is treated as Near-Threatened because of the potential overexploitation of natural populations by mushroom gatherers (Antonín 2006).

***Mel-10* (*M.importuna* M. Kuo, O‘Donnell & T.J. Volk; Fig. 5B).** Mel-10 is a newly recognized species for the Czech Republic, although our results show that it has already been a part of the Czech mycobiota for decades. In total, 70 *Morchella* cultures or exsiccated specimens (the oldest one was collected in 1950) previously morphologically identified mostly as *M.pragensis* (18 specimens), *M.conica* (12 specimens), or *M.elata* (nine specimens), or designated just as “black mulch morel” (18 specimens) were determined as Mel-10. The ascomata of the Czech collections were extremely variable in shape. The specimens that were examined were collected from mid-April to mid-May. It is noteworthy that *M.importuna* is probably a later synonym for several validly published names, e.g., *M.elata*, *M.hortensis* Boud. or *M.vaporaria* Brond. However, the interpretation of these names is unresolved and the name *M.importuna* was therefore provisionally retained for Mel-10 by Richard et al. (2015). Among Czech mycologists and morel hunters, the species is often treated as *M.pragensis*. This name was published twice (Smotlacha 1947, 1952) on the basis of collections from the surroundings of Prague, firstly without the Latin diagnosis, secondly without the holotype being indicated (which, nevertheless, was not necessary before 1958) and as two forms without the nominate form being specified. The nomenclatural errors were later corrected and the neotype was assigned by Moravec (1970). However, sequencing of the neotype has not been successful yet; its identity needs to be determined in future studies. Despite the formal errors, *M.pragensis* became widely known among the public and the name has been commonly used for various collections from anthropogenic habitats, particularly ruderal places such as waste dumps and debris after demolition, but also gardens, yards, ornamental beds with bark mulch, etc.

*Morchellaimportuna* was described from the USA in 2012 (Kuo et al. 2012) and it was hypothesized as originating in western North America, from where it has spread in association with horticulture and silviculture (Taşkın et al. 2010), but it has also been reported from Germany, Poland, Finland, France, Switzerland, Spain, Turkey, Cyprus, Israel, Canada, and China (Taşkın et al. 2010, 2012; Du et al. 2012a, 2012b; Richard et al. 2015; Loizides et al. 2016). The species appears to be a saprotroph (Mann and Mann 2014), and therefore it can be cultivated artificially (Du et al. 2015). *Morchellaimportuna* is also known as a facultative post-fire species (Clowez 2012; Du et al. 2012a; Loizides 2017), and recent research has shown that it can even be grown on fire-treated fields (Li et al. 2017). A morphological description of *M.importuna* was given by Clowez (2012; under the name *M.vaporaria*) and Kuo et al. (2012). It may be difficult to distinguish *M.importuna* from other species in the Elata Clade morphologically. However, the best clue for its identification may be its occurrence in urban habitats in combination with regularly laddered, vertically oriented pits and ridges on ascomata (Kuo et al. 2012; Mann and Mann 2014). Both in its presumed native distribution area and in the Czech Republic it occurs in various urban habitats, particularly woodchip or mulch beds (Kuo et al. 2012); therefore, it is sometimes called the “mulch morel” (Mann and Mann 2014). But it is also frequently found in the yards of houses, in masonry, dumps of rubble, sand, wood or bark, and one Czech specimen was collected in an old fire pit. Only occasionally was this species found in semi-natural habitats such as forests, along forest paths, or in meadows or town parks.

***Mel-13/26* (*M.deliciosa* Fr.; Fig. 5C).** Eight *Morchella* cultures or fungarium specimens phenotypically identified mostly as *M.conica* (four specimens) clustered with both Mel-13 (no Latin binomial) and Mel-26 (*M.deliciosa*). While the former species was distinguished by O’Donnell et al. (2011), the latter one was delimited by Taşkın et al. (2010), but without Mel-13 being used in their analysis. It was only later that Taşkın et al. (2012) analyzed both species together and revealed the paraphyly of Mel-13 because of the exclusion of Mel-26 from the clade. The separation of Mel-26 can therefore be considered inappropriate and both species were combined by Du et al. (2015) as Mel-13/26 (*M.deliciosa* being the only known Latin binomial). Our results confirm this treatment unambiguously. Mel-13 has so far only been reported from Asia (China, India, and Turkey; O’Donnell et al. 2011; Du et al. 2012a; Taşkın et al. 2012; Richard et al. 2015), while Mel-26 has, in addition to Turkey (Taşkın et al. 2010, 2012), also been reported from some European countries (France, Poland, and Sweden; Clowez 2012, as several varieties of *M.conica*; Taşkın et al. 2012; Richard et al. 2015; Baran and Boroń 2017). The Czech specimens that were examined were collected in mid-April in mixed forests, mainly under *Fraxinusexcelsior*, *Picea* sp., and *Pinus* sp. Other nearby trees or shrubs were *Quercus* sp., *Larixdecidua*, *Fagussylvatica*, and *Sambucusnigra*. Other authors (Taşkın et al. 2010, 2012; Clowez 2012) mainly reported associations with conifers (*L. decidua*, *Piceaabies*, *Pinus* sp.), while Baran and Boroń (2017) also observed *Abiesalba*, *Tiliaplatyphyllos*, *Acerpseudoplatanus*, and *Euonymusverrucosa* at the localities where *M.deliciosa* was collected.

***Mel-15/16* (*M.angusticeps* Peck / *M.eximioides* Jacquet.; Fig. 5D).** Nine *Morchella* cultures or fungarium specimens previously morphologically recognized mostly as *M.conica* (six specimens) clustered with Mel-15 (*M.angusticeps*) or Mel-16 (*M.eximioides*). Both species were originally delimited by O’Donnell et al. (2011) on the basis of a sample set of seven eastern North American (Mel-15) and four Scandinavian specimens (Mel-16), which exhibited stable polymorphism (i.e., synapomorphies) at *EF-1α* and *RPB2*, but not at LSU and *RPB1*. Nevertheless, the Czech samples share synapomorphies with both of the species and form a basal lineage to them (Fig. 1). The Czech Mel-15/16 specimens fulfill the criterion of monophyly and the genealogical criterion (Suppl. material 3, Fig. S1) and thus could be distinguished as a separate phylospecies according to O’Donnell et al. (2011). However, the three lineages are distinguishable only by *EF-1α* and *RPB2* (Suppl. material 3, Fig. S1, Suppl. material 5, Table S3), and the total detected genetic distance of the Czech specimens from Mel-15 and Mel-16 is eight and 15 SNP’s, respectively. Moreover, variation within branches is higher than that among branches at some loci and the split into three lineages therefore may be caused, hypothetically, by geographic variation and limited sampling. Consequently, we prefer not to assign the Czech lineage as a new phylospecies before additional (e.g., phenotypic) data prove its distinctness.

It is generally supposed that Mel-15 is endemic to eastern North America (O’Donnell et al. 2011; Kuo et al. 2012; Richard et al. 2015), while Mel-16 has been reported from Northern Europe and China (O’Donnell et al. 2011; Du et al. 2012a). This early fruiting species (recent collections were made in approximately mid-April) appeared in both (semi-)natural and anthropogenic habitats. Five specimens were found in deciduous forests, a town park or an old orchard. Two other specimens were collected in the vicinity of paper mills on paper or wood waste. Two specimens (collected in different years) were found by a sedimentation basin of a heating plant. The species grew together with various deciduous trees and shrubs, including *Prunus* spp., *Fraxinus* sp., *Acer* sp., *Populus* sp., *Crataegus* sp., *Cornussanguinea*, *Betula* sp., or *Salix* sp. Du et al. (2012a) also reported the association of Mel-16 with *Picea* sp.

***Mel-19* (*M.eohespera* Beug, Voitk & O‘Donnell; Fig. 5E).** Forty-three *Morchella* cultures or fungarium specimens morphologically identified mostly as *M.conica* (24 specimens), *M.elata* (seven specimens), or *M.pragensis* (six specimens) were determined as Mel-19 (*M.eohespera*; an older name is possibly *M.norvegiensis* Jacquet.; see Voitk et al. 2016b). Although Mel-19 was previously recorded from the Netherlands, Sweden, Switzerland, China, and the USA (O’Donnell et al. 2011; Du et al. 2012a; Taşkın et al. 2012; Beug and O’Donnell 2014; Richard et al. 2015), it was only after the collections from Canada that the Latin binominal was given to this phylogenetic species (Beug and O’Donnell 2014; Voitk et al. 2016a). A morphological description of this species is available in Voitk et al. (2016a). In the Czech Republic, *M.eohespera* appeared from mid-April to mid-May, but the most recent collections were mostly made around mid-April. The specimens that were examined were collected in a variety of habitats including different types of forests (where they often occurred along roads, on deposits of wood on wood waste, on hillsides, and in river or creek valleys), in gardens, old yards, rubble sites, railway stations, and other urban habitats, sandstone quarries, a brick factory, a meadow, and also on bark mulch. Voitk et al. (2016a) also reported the occurrence of this species both in natural habitats and at sites significantly affected by human activities. The Czech collections of *M.eohespera* were frequently found together with *Populus* spp. (mostly *P.tremula*), *Betulapendula*, *Piceaabies*, or *Pinussylvestris*, while other nearby trees were *Salix* spp., *Fagussylvatica*, *Fraxinusexcelsior*, *Quercus* sp., and *Malusdomestica*. The species can also be found in association with other trees such as *Alnus* sp., *Corylus* sp., or *Abies* sp. (Du et al. 2012a ; Voitk et al. 2016a).

***Mel-20/34* (*M.purpurascens* (Boud.) Jacquet; Fig. 5F).** One culture and 21 fungarium specimens previously morphologically identified mostly as *M.conica* (11 specimens) or *M.elata* (six specimens) were determined as Mel-20, corresponding to *M.purpurascens*, or as Mel-34, which lacks a Latin binominal. Mel-20 was originally distinguished by O’Donnell et al. (2011) as a sister lineage to Mel-19 (*M.eohespera*) with a very low bootstrap support at most of the loci (< 50%) and 93% support at *EF-1α*, which contains almost all of the few apomorphies that distinguish the two lineages. Later, Du et al. (2012a) distinguished Mel-34 on the basis of a single specimen from China that is almost identical to Mel-20 at all loci except *RPB1*, which provided an almost identical sequence to Mel-23/24/31/32 (*M.pulchella*; Suppl. material 3, Fig. S1, Suppl. material 5, Table S3). Therefore, we propose merging Mel-34 with Mel-20 (*M.purpurascens* being the only known binomial) provisionally until more collections are made and this extraordinary pattern is confirmed. The distinctness of Mel-19 and Mel-20 is still clear after the inclusion of the Czech samples, although the difference is very small, the latter branch is poorly supported (posterior probability 0.74) and distinction of the two lineages may thus be an artifact of anagenesis in the *EF-1α* gene. Not surprisingly, distinguishing between these species on the sole basis of ITS is tricky, as they differ in a single SNP. Moreover, this polymorphism is not stable across the whole ranges of these species, but it was stable for all of the Czech specimens that were studied. We therefore considered it as diagnostic in ITS-based determinations.

Mel-20 (*M.purpurascens*) is known from France, Scandinavia, Turkey, China, and Taiwan (Taşkın et al. 2010, 2012; O’Donnell et al. 2011; Clowez 2012, as a variety of *M.conica*; Du et al. 2012a; Richard et al. 2015). The ascomata of the Czech specimens were collected between late April and mid-May, both in anthropogenic habitats (railways, roadsides, gardens, and also in a junkyard, places with deposits of various materials such as rubble, sand, wood, or pure brick clay, between stones or even on concrete) and in forests, and co-occurred with both conifers (often growing under *Picea* sp. or *Pinus* sp.) and deciduous trees and shrubs (*Quercus* sp., *Prunusdomestica*, *Betula* sp., *Crataegus* sp., *Populus* sp., *Salix* sp.). Other authors also reported the frequent co-occurrence of Mel-20 with conifers such as *Abies* sp., *Pinus* sp., or *Cedrus* sp. and also with *Populus* sp. or *Quercus* sp. (Taşkın et al. 2010, 2012 ; Clowez 2012 ; Du et al. 2012a).

***Mel-23/24/31/32* (*M.pulchella* Clowez & F. Petit; Fig. 5G).** Twenty-five specimens (including two samples from Slovakia) originally determined mostly as *M.conica* (10 specimens), *M.elata* (four specimens), or *M.pragensis* (five specimens), grouped together with Mel-23, Mel-24, Mel-31, and Mel-32. Mel-23 (no Latin binominal) and Mel-24 (*M.septentrionalis* M. Kuo, J.D. Moore & Zordani) were originally distinguished by O’Donnell et al. (2011) on the basis of three Scandinavian specimens and one specimen (plus five additional that were not shown) from the eastern USA and Canada, respectively. Mel-31 (*M.pulchella*) was delimited in parallel by Taşkın et al. (2010) without the inclusion of Mel-23 and Mel-24 in the analyses and has so far been reported from China, Pakistan, Turkey, and France (Taşkın et al. 2010, 2012; Du et al. 2012a; Richard et al. 2015; Badshah et al. 2018). Mel-32 (*M.conifericola* Taşkın, Büyükalaca & H.H. Doğan) was later distinguished by Taşkın et al. (2012) in Turkey, although the clade was poorly supported (BS = 59 for concatenated data and < 50% for individual loci). Moreover, the latter study revealed the paraphyly of Mel-31 as a result of the exclusion of Mel-24, and also very low support (if any) for each of the four species. These facts were confirmed by Richard et al. (2015) and our own data (Fig. 1). Therefore, we suggest combining the four formerly delimited species and to treat them as one, with the oldest known Latin binomial being *M.pulchella*, and *M.septentrionalis* and *M.conifericola* being later synonyms. Two specimens corresponding to the Mel-23/24/31/32 lineage were also reported from India (Du et al. 2012b). The degree of endemism, therefore, appears to be overestimated and the recorded variation may be attributed to phylogenetically young mutations and sometimes to intraspecific geographic variability. The Czech specimens were collected from mid-April to early May, mostly in forests (often along roads or forest edges), but also along railways or in sandstone quarries. A special collection site was a surface coal mine where a stable population of morels was visited in several successive years in the 1970s. Ascomata were mostly found under deciduous trees such as *Populustremula*, *Carpinusbetulus*, *Betulapendula*, or *Fraxinus* sp., while other nearby trees were *Tilia* sp., *Salixcaprea*, *Quercus* sp., and occasionally conifers (*Pinus* sp., *Picea* sp.).

***Mel-39* (newly designated phylogenetic species; Fig. 5H).** Two cultures and four exsiccated specimens formed a well-separated and well-supported lineage sister to Mel-10 (*M.importuna*). Although closely related to Mel-10, this lineage differs at each of the loci that were studied by three (LSU) to 18 (*EF-1α*) synapomorphic SNP’s (59 SNP’s in total; Suppl. material 5, Table S3). This lineage is identical to the New-2 clade sensu Du et al. (2012b), which was reported from China and Germany based on ITS only; however, it has not been definitely distinguished as a phylospecies until now. Beside the significant genetic differences and genealogical concordance, Mel-10 and Mel-39 differ in several phenotypic traits, particularly on sclerotia under experimental cultivation. Whereas the sclerotia of Mel-39 are very tiny (mostly not bigger than 1 mm), spherical, not coalescing, dark, red-brown, mature sclerotia of *M.importuna* are of a light color varying from that of a walnut shell to somewhat orange, and coalesce into big hardened bodies of an irregular shape that are up to several centimeters long (Petrželová unpublished data). Nevertheless, formal taxonomic treatment needs to be based on extensive phenotypic analyses and the study of the type material of related taxa, and cannot be performed at this stage. The species was mostly collected on bark mulch, mostly in late April or early May, i.e., similarly to or slightly earlier than Mel-10.

***Mes-4* (*M.americana* Clowez & Matherly; Fig. 5I).** One *Morchella* strain maintained as an axenic culture and previously morphologically determined as *M.esculenta* was identified as Mes-4 (Fig. 2; Suppl. material 2, Table S2). This species was described under several binominals (Clowez 2012; Kuo et al. 2012), and *M.americana* was selected by Richard et al. (2015) as the most appropriate among the priority names. However, Clowez (2012) used the name *M.rigida* (Krombh.) Boud. for the French specimens that Richard et al. (2015) found as conspecific with Mes-4. If this name of Krombholz (a 19^th^-century Prague mycologist) was used correctly, *M.rigida* (basionym M.conicavar.rigida; Krombholz 1831–1834) is probably the oldest name for the species. Nevertheless, we follow the latest treatment of Richard et al. (2015) for now.

*Morchellaamericana* appears to be native to North America, where it is the most widely distributed Esculenta clade species (O’Donnell et al. 2011; Du et al. 2012b; Kuo et al. 2012; Richard et al. 2015). To date, it has also been reported from France, Spain, Turkey, and China (Taşkın et al. 2010, 2012; Du et al. 2012b; Richard et al. 2015), and this is the first record of the species for the Czech Republic (not considering Krombholz’s collections). In North America it mostly co-occurs with *Fraxinus* spp., *Ulmusamericana*, *Populus* spp., *Platanusoccidentalis*, *Acer* sp., or *Quercus* spp., but it can also be found in old apple orchards and occasionally together with conifers (Kuo et al. 2012; Richard et al. 2015). Its association with *Buxussempervirens* has also been reported (Clowez 2012; Richard et al. 2015). It has been suggested that *M.americana* has only recently been introduced to Europe, as most records come from sites with a strong anthropogenic influence, especially from hybrid poplar plantations (Richard et al. 2015). The Czech specimen, nevertheless, occurred in the Žebračka National Nature Reserve, i.e., a site with one of the most valuable natural alluvial forests in the Czech Republic. Moreover, if Krombholz’s specimens originating from Czech Republic were really identical with Mes-4, human-mediated introduction from North America would be rather unlikely. The studied specimen was found under young *Populustremula* trees at the beginning of May.

***Mes-5* (Fig. 5J).** Nineteen *Morchella* cultures or exsiccated specimens (including two samples from Slovakia) that had previously been phenotypically determined differently, mostly as *M.vulgaris* (eight specimens) or *M.esculenta* (three specimens), but also as a variety of species of black morels, were identified as Mes-5 (no Latin binominal). Although the multi-gene approach was only used for two specimens, no clear polymorphism was found at ITS among our Mes-5 accessions, whereas two SNP’s were observed between Mes-5 and the sister Mes-17 (*M.dunensis*). Therefore, identification based on ITS should be sufficient. Mes-5 has so far been found in Denmark, France, and Norway (O’Donnell et al. 2011). The Czech specimens were mostly collected from mid-April to early May, often in gardens (also on bark mulch) or in forests, but some collections were also made in a park, an orchard, a meadow and a waste dump near a summer cottage. Ascomata of this species were often found under fruit trees (*Malusdomestica*, *Pyrus* sp., *Prunuspersica*), and Rosaceae shrubs (e.g., *Crataegus* sp.); other nearby trees that were recorded were *Fraxinusexcelsior*, *Acerpseudoplatanus*, *Populus* sp., *Robiniapseudoaccacia*, and *Piceaabies*, and in Slovakia also *Swida* sp. and *Pinusnigra*.

***Mes-8* (*M.esculenta* (L.) Pers.; Fig. 5K).** Forty-seven *Morchella* cultures or exsiccated specimens, (including three samples from Slovakia) previously morphologically determined mostly as *M.esculenta* (29 specimens) or less frequently as *M.crassipes* (eight specimens) or *M.vulgaris* (six specimens), were identified as Mes-8 (corresponding to *M.esculenta*). *Morchellaesculenta* is the common and widely distributed European morel species recorded from the Czech Republic, Poland, Germany, Switzerland, France, Spain, Belgium, the Netherlands, Norway, Sweden, and Turkey (Taşkın et al. 2010, 2012; O’Donnell et al. 2011; Clowez 2012; Du et al. 2012b; Richard et al. 2015; Baran and Boroń 2017) but also from China (Du et al. 2012a). The Czech collections were mostly obtained from mid-April to early May, occasionally up to mid-May, mostly in (semi-)natural habitats in deciduous (including floodplain) forests and shrubs, less often in limestone quarries, old orchards, parks, or gardens (here also on bark mulch). A special collection site was the edge of a reed bed. Uncommonly, *M.esculenta* was found in ruderal or urban habitats such as the yards of buildings, a rubble site or even the concrete floor of a woodshed. *M.esculenta* has been found in association with a variety of deciduous wood species. The Czech collections were more frequently collected under *Fraxinus* sp. (often *F.excelsior*), *Crataegus* sp., *Prunus* spp. (especially *P.spinosa*, *P.domestica*, and *P.avium*), *Quercus* spp., and *Acer* spp. Other nearby trees were *Alnus* sp., *Carpinusbetulus*, *Populustremula*, *Betula* sp., *Salixcaprea*, *Malusdomestica*, *Aesculushippocastanum*, *Robiniapseudoaccacia*, and occasionally conifers such as *Picea* sp., *Larix* sp., *Pinussylvestris*, or *Thuja* sp. According to other authors, *M.esculenta* can also grow under *Ulmus minor, Malussylvestris, Cydonia oblonga, Mahonia* sp., or, rarely, Cupressaceae species (Clowez 2012) or *Abies* sp. (Taşkın et al. 2012).

### Mulch morels

What are known as the “mulch morels” represent a specific ecological group of morels that occur massively in newly created ornamental beds with bark mulch, mostly in gardens or around construction zones and newly built houses. On the basis of our observations, the macromorphological variation both within and among populations of mulch morels is remarkable, sometimes to such an extent that it brings to mind the variation among species. Therefore, we aimed at an estimation of the number of species within and among neighboring localities. However, with the only exception of one site with Mel-10 (*M.importuna*) and Mel-39, all the samples from the same ornamental bed belonged to the same species (Mel-10; Suppl. material 1, Table S1). Nevertheless, among the total of 48 specimens that originated from different localities with bark mulch, five *Morchella* species were recognized. A total of 36 specimens were determined as Mel-10 (*M.importuna*), five as Mel-39, four as Mes-5, two as Mel-19 (*M.eohespera*), and one as Mes-8 (*M.esculenta*; Suppl. material 2, Table S2).

## Conclusions

*Morchella* taxonomy may give the impression of being opaque for many field mycologists. Much of the confusion appears to stem from the excessive or inappropriate over-splitting of some phylogenetic clades into smaller and poorly supported subclades and from the apparent lack of consensus on taxonomical principles. Therefore, we propose five criteria for distinguishing the new phylospecies in *Morchella*: the criterion of monophyly, the genealogical criterion, the criterion of distinctness, the criterion of minimal sampling, and the criterion of polygenic differentiation. Surely, none of them absolutely reflects natural processes related to speciation and DNA sequence evolution (Leliaert et al. 2014) and each of them can be modified in specific cases. Nevertheless, we believe that the application of these five criteria in distinguishing new phylospecies could prevent further confusion in the molecular taxonomy of morels, although some phylospecies may remain overlooked and undetected. Our approach, therefore, is conservative and pragmatic, aiming at the practical usage of taxonomy, rather than at identification of all possibly existing small evolutionary units. It is stressed that in this study we rely on molecular phylogenetics only. The most straightforward method for recognition of the species would be one based on phenotypic traits, which should also serve as a support for the delimitation of species. Considering that phenotypic traits are often highly influenced by plasticity (i.e. environmental conditions) and/or intraspecific variability, identification of discriminating macro-and microscopic characters that correspond to the phylogenetic species will be the greatest challenge. This is, nevertheless, necessary in order to link the various phylospecies to the appropriate binomials, especially the old names whose type material is not available for molecular analyses. Integrative studies combining both phenotypic and molecular methods will, hopefully, result in a clearer, phylogeny-based and sustainable *Morchella* taxonomy.

## Supplementary materials

Supplementary material 1 List of localities of mulch morels from which more than one ascoma was analyzed Data type: occurrence This dataset is made available under the Open Database License (http://opendatacommons.org/licenses/odbl/1.0/). The Open Database License (ODbL) is a license agreement intended to allow users to freely share, modify, and use this Dataset while maintaining this same freedom for others, provided that the original source and author(s) are credited.Irena Petrželová, Michal Sochor

Supplementary material 2 List of analysed *Morchella* specimens with collection data, determination and Genbank accession numbers Data type: species data This dataset is made available under the Open Database License (http://opendatacommons.org/licenses/odbl/1.0/). The Open Database License (ODbL) is a license agreement intended to allow users to freely share, modify, and use this Dataset while maintaining this same freedom for others, provided that the original source and author(s) are credited.Irena Petrželová, Michal Sochor

Supplementary material 3 Single-gene bayesian trees; posterior probabilites shown above branches Data type: phylogenetic data This dataset is made available under the Open Database License (http://opendatacommons.org/licenses/odbl/1.0/). The Open Database License (ODbL) is a license agreement intended to allow users to freely share, modify, and use this Dataset while maintaining this same freedom for others, provided that the original source and author(s) are credited.Irena Petrželová, Michal Sochor

Supplementary material 4 Supplementary data Data type: measurement This dataset is made available under the Open Database License (http://opendatacommons.org/licenses/odbl/1.0/). The Open Database License (ODbL) is a license agreement intended to allow users to freely share, modify, and use this Dataset while maintaining this same freedom for others, provided that the original source and author(s) are credited.Irena Petrželová, Michal Sochor

Supplementary material 5 Matrices of genetic distances detected at each locus for selected accessions. A. ITS, B. LSU and EF-1a, C. RPB1 and RPB2 Data type: molecular data This dataset is made available under the Open Database License (http://opendatacommons.org/licenses/odbl/1.0/). The Open Database License (ODbL) is a license agreement intended to allow users to freely share, modify, and use this Dataset while maintaining this same freedom for others, provided that the original source and author(s) are credited.Irena Petrželová, Michal Sochor
